# Beyond QTL Cloning

**DOI:** 10.1371/journal.pgen.1001197

**Published:** 2010-11-11

**Authors:** Jill T. Anderson, Thomas Mitchell-Olds

**Affiliations:** Institute for Genome Sciences and Policy, Department of Biology, Duke University, Durham, North Carolina, United States of America; The University of North Carolina at Chapel Hill, United States of America

Natural selection sculpts phenotypes to enable adaptation to local conditions. Biologists have long been interested in the phenotypic and molecular response to selection, but we still know little about the genetic basis of adaptation. With continuing improvements in molecular technologies, we now have the tools to investigate the genes that underlie ecologically important traits. Assessing the geographic distribution of allelic variation at these loci can point to the agents of selection, if different alleles are advantageous in contrasting environmental conditions [1]–[3]. In theory, predictable patterns of clinal variation in allele frequencies can arise from divergent selection across environmental gradients [1], although demographic factors may complicate identification of adaptive changes [4], [5].

Few studies have linked geographic variation in allele frequencies with variation in phenotypes (but see [6]–[8]). Studies of clinal variation are especially powerful when a causative gene underlying a key phenotype has already been identified. For example, geographic clines in flowering time are associated with variation at flowering time genes in the model species *Arabidopsis thaliana* [2], [3]. Many studies focus on traits that vary on a broad geographic scale, for which latitude, longitude, or elevation can be used as a proxy for environment. Other environmental conditions are more difficult to measure at the landscape level, and have therefore often been ignored in studies of the geographic distribution of genetic and phenotypic variation.

In this issue of *PLoS Genetics*, Baxter et al. [9] present an elegant study of the geographic variation in salinity tolerance, and allelic variation at the sodium transport gene *AtHKT1;1* in European populations of *A. thaliana*. In a laboratory experiment, Baxter and colleagues grew *Arabidopsis* accessions in non-saline soil (without elevated Na^+^) and found substantial variation in the accumulation of sodium in leaf tissue, which indicates tolerance to saline soils. Furthermore, foliar Na^+^ of these genotypes decreased with distance from the collection site to nearby regions with saline soils [9]. This geographic pattern suggests that the phenotype (salinity tolerance) could be a direct evolutionary response to a stressful environmental condition (saline soils).

A genome-wide association study revealed a significant association between the *AtHKT1;1* candidate gene and foliar Na^+^ accumulation [9], corroborating results of previous studies [10], [11]. Baxter et al. [9] conducted additional crosses between accessions with high and low foliar Na^+^, in conjunction with gene expression studies, and found that elevated salinity tolerance is associated with hypofunctional alleles of *AtHKT1;1*. These molecular data can be brought to bear on the geographic cline in salinity tolerance: accessions with a hypofunctional allele at *AtHKT1;1* occur in areas where the soil is likely to be saline.

Baxter and colleagues [9] conclude that the geographic cline in *AtHKT1;1* is an adaptive response to variation in soil salinity. In addition, human disturbance could contribute to this allelic cline, as the genotype associated with salt tolerance is distributed primarily near major ports and could have been dispersed along shipping lanes (see Figure 1 in [9]). Additional sampling, as well as ecological studies and whole-genome analysis of SNP geography, may clarify the relative importance of natural selection and possible human-mediated gene flow in the geographic distribution of alleles at *AtHKT1;1.*

## What Comes after QTL Cloning?

For the past decade, QTL (quantitative trait loci) cloning has been the rate-limiting step for many laboratories. Now, improvements in technology and resources are enabling advances such as the current study, and biologists can turn to functional elucidation of newly identified genes and ecological analyses of plants in their environments. As the field matures, molecular biologists and ecologists should work together towards field studies of allelic adaptation to local environments. Experimental plants can be grown under ecologically relevant conditions, e.g., in soil of different salinities collected from multiple sites, or in common garden field experiments at high and low salinity sites. Quantification of traits and fitness components under natural conditions is essential for measuring selection on phenotypes and on alleles at candidate genes. In this instance, field studies could test whether the hypofunctional allele at *AtHKT1;1* increases salinity tolerance and reproductive fitness in saline soils, and whether it diminishes fitness in non-saline soils. These questions determine whether local adaptation is due to tradeoffs in performance between different environments (a home genotype advantage), which can maintain genetic variation within species [12].

Determining the mechanistic basis of geographic clines will be considerably more complicated for studies of quantitative traits controlled by multiple loci. Nature is far more complex than laboratory conditions, and candidate genes identified in the lab might not be associated with traits in the field [13]–[15]. Here, Baxter et al. have made substantial progress identifying the causal gene underlying tolerance to a stressful environment, and have demonstrated that stress tolerance is associated with specific features of the landscape (i.e., proximity to saline soils). Future integrative studies combining techniques from molecular genetics, ecology, and evolutionary biology can begin to unveil the genetic basis of adaptation in model and non-model species alike.
